# Chinese expert consensus on diagnosis and management of gastroesophageal reflux disease in the elderly (2023)

**DOI:** 10.1002/agm2.12293

**Published:** 2024-04-12

**Authors:** Liu Fangxu, Li Wenbin, Zhang Pan, Chen Dan, Wu Xi, Xu Xue, Shi Jihua, Luo Qingfeng, Xu Le, Zheng Songbai, Chinese Geriatrics Society, Chen Minhu, Chen Xinyu, Ding Shigang, Ding Xiping, Hou Xiaohua, Huang Xiaoli, Ji Rui, Jiang Hua, Li Jingnan, Li Peng, Liu Ruixue, Liu Shixiong, Liu Zhijun, Long Limin, Miao Lin, Ruan Jigang, Shao Yun, Shi Liping, Sun Xiaohong, Tang Chengwei, Wan Jun, Wang Fengyun, Wang Gangshi, Wang Jingtong, Wang Ruiling, Wen Shujun, Wu Jimin, Wu Jing, Xiao Yinglian, Xu Lishu, Xu Shiping, Yao Jianfeng, Yao Ping, Yin Tiejun, Yu Pulin, Zhang Hangxiang, Zhang Weisan, Zhang Yijun, Zhao Li, Zhong Bihui, Zhou Bingxi, Zhou Liya, Zou Duowu

**Affiliations:** ^1^ Department of Gastroenterology, Beijing Hospital, National Center of Gerontology, Institute of Geriatric Medicine Chinese Academy of Medical Sciences Beijing China; ^2^ Department of Geriatrics Huadong Hospital Affiliated to Fudan University Shanghai China

**Keywords:** aged, expert consensus, gastroesophageal reflux

## Abstract

Gastroesophageal reflux disease (GERD) in the elderly is characterized by atypical symptoms, relatively severe esophageal injury, and more complications, and when GERD is treated, it is also necessary to fully consider the general health condition of the elderly patients. This consensus summarized the epidemiology, pathogenesis, clinical manifestations, and diagnosis and treatment characteristics of GERD in the elderly, and provided relevant recommendations, providing guidance for medical personnel to correctly understand and standardize the diagnosis and treatment of GERD in the elderly.

## Graphical abstract

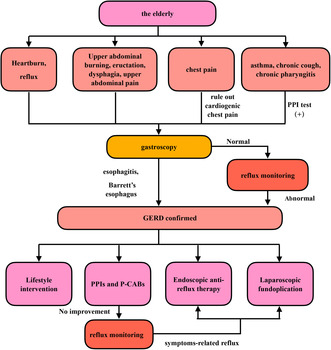

## 1 INTRODUCTION

Gastroesophageal reflux disease (GERD) is a common clinical disease, and its prevalence increases with aging. At the end of 2020, the elderly population aged 60 and above in China had reached 264 million, accounting for 18.7% the total population.^1^ As China gradually enters an aging society, the number of GERD elderly patients is also increasing year by year. GERD in the elderly, compared with the non‐elderly, has such characteristics as atypical symptoms, severe esophageal injury, and more complications, which require special attention. At present, there are no specific guidelines for or expert consensus on standardizing the diagnosis and treatment of GERD in the elderly population within and outside China. Therefore, based on *Chinese expert consensus of gastroesophageal reflux disease in 2020* formulated by the Chinese Society of Gastroenterology, the Elderly Digestion Group of the Chinese Society of Geriatrics organized a consensus opinion expert committee composed of relevant experts in the field in China. The working group searched such databases as Medline and Wanfang Data Knowledge Service Platform and formulated a draft of this consensus. Subsequently, the expert committee held multiple rounds of discussion and voting until a consensus was reached. The recommendation of voting opinions is divided into six grades: strongly recommended (A+); recommended with a few reservations (A); recommended with many reservations (A−); not recommended with many reservations (D); not recommended with a few reservations (D−); not recommended at all (D+). The corresponding evidence is divided into four levels: high quality, indicating that further research cannot change the credibility of the effectiveness evaluation results; medium quality, indicating that further research is likely to affect the credibility of the effectiveness evaluation results and may change the evaluation results; low quality, indicating that further research is highly likely to affect the credibility of the effectiveness evaluation results and is likely to change the evaluation results; extremely low quality, indicating that all effectiveness evaluation results are highly uncertain. This consensus contains a total of 26 consensus opinions.

## 2 EPIDEMIOLOGY OF GERD IN THE ELDERLY

A meta‐analysis of 79 global studies showed that the global average prevalence of adult GERD was 13.3%, with great differences in prevalence among different regions and countries, with an average prevalence of 10.0% in Asia; the analysis also showed that the prevalence of GERD increased with aging, with a prevalence of 17.3% for people aged ≥50 years and 14.0% for those aged <50 years.^2^ A survey in Japan in 2015 showed that the prevalence of GERD among elderly people aged 65 years and above was 17.5%, while the prevalence among people aged 20–64 years from the same community was 10.8%.^3^ The study results of Songbai et al.^4^ in China showed that the detection rate of endoscopic reflux esophagitis in the elderly group (≥60 years old) was 8.9%, higher than that in the non‐elderly group (4.3%). The symptoms of GERD in the elderly are milder, but the histological damage is more severe; therefore, it is possible that many studies underestimated the rate of GERD in the elderly population.^5^ A study from the United States in 2016 that included 35 million patients during a 10‐year period (2001–2010) showed that the incidence of GERD, Barrett's esophagus, and esophageal cancer all increased with aging.^6^

**Consensus opinion 1:** The prevalence of GERD increased with aging (recommendation grades: A+, 86%: A, 14%. Evidence level: High quality).

## 3 PATHOGENESIS OF GERD IN THE ELDERLY

GERD patients often had decreased esophageal clearance ability, while the proportion of esophageal motility disorders and ineffective esophageal contractions in the elderly increased.^7^, ^8^ Other study results showed that with aging, the conduction velocity of esophageal peristalsis decreased in healthy volunteers and GERD patients after normal swallowing.^9^ The success rate of wet swallowing in GERD patients decreased.^10^ Transient lower esophageal sphincter relaxation (TLESR) was an important mechanism of GERD, but the resting pressure of the lower esophageal sphincter (LES) was not related to aging.^7^, ^8^, ^9^ Other study results showed that the upper esophageal sphincter (UES) pressure in the elderly decreased,^8^, ^11^ and the resting pressure of UES was negatively correlated with aging.^10^

The decrease in esophageal clearance ability in the elderly was also related to a decrease in salivary and bicarbonate secretion, while salivary secretion was an important pre‐epithelial factor for the complete esophageal mucosal barrier. Salivary secretion decreased with aging, with about 25% of the elderly suffering from dry mouth. Compared with the young control group, middle‐aged and elderly subjects (>55 years old) showed a significant decrease in salivary and bicarbonate response to esophageal acid perfusion. A questionnaire study with 531 patients included showed that patients with hyposalivation significantly increased in age and had severe dry mouth and gastroesophageal reflux‐like symptoms, and multivariate analysis results showed that advanced age and severe gastroesophageal reflux‐like symptoms were independently associated with hyposalivation.^12^

Esophageal hiatus hernia was an important factor for the occurrence of gastroesophageal reflux,^13^, ^14^ the size of which was related to the severity of GERD,^15^ and which with a diameter greater than 3 cm was associated with severe reflux esophagitis.^16^, ^17^ The incidence of esophageal hiatus hernia increased with aging, and the meta‐analysis results showed that esophageal hiatus hernia was associated with age above 50 years, with an OR of 2.71.^18^ Other study results showed that 60% of people over 60 years had esophageal hiatus hernia.^19^

Multiple chronic diseases that were often comorbid in the elderly were associated with the occurrence of GERD. The prevalence of Parkinson's disease increased with aging, with a prevalence of approximately 1% among people aged 60 years and above.^20^ Gastrointestinal nerve cells could produce dopamine, which could regulate gastrointestinal motility.^21^ Parkinson's disease could cause abnormal dopamine secretion, leading to severe gastrointestinal dysfunction. Gastrointestinal symptoms were developed in 60% to 80% of Parkinson's disease patients, some of which might appear 5 years earlier than typical motor symptoms.^22^ Parkinson's disease was associated with the occurrence of GERD,^23^ early study results showed that the prevalence of heartburn in Parkinson's disease patients was twice that in the control group,^24^ and the aforementioned conclusion could also be reached in another questionnaire study.^25^ However, in the study by Edwards et al.,^26^ there was no such difference. Similarly, in a study with 329 patients included in 2015, GERD was not shown to be associated with Parkinson's disease.^27^

Type 2 diabetes mellitus was common in the elderly, and the prevalence of GERD in type 2 diabetes mellitus patients was higher than that in the general population,^28^ which might be associated with the abnormal esophageal motility caused by neuropathy in type 2 diabetes mellitus patients.^29^, ^30^ The proportion of GERD was significantly increased in the diabetic patients with neuropathy.^28^ In addition, the proportion of asymptomatic gastroesophageal reflux was high in the diabetic patients.^31^

Elderly patients with GERD generally experienced autonomic nervous system dysfunction characterized by depression, anxiety, and decreased vagal excitability, and the degree of negative emotions was significantly associated with the severity of GERD symptoms.^32^ The incidence of anxiety and depression in the elderly patients with GERD was as high as 44.3%, which was higher than that in the non‐elderly patients with GERD (34.8%).^33^ Another study with 367 elderly patients with GERD and 209 non‐elderly patients with GERD included showed that the incidence of anxiety and depression in the elderly patients with GERD was as high as 64.03%, higher than that in the non‐elderly patients with GERD (40.19%).^34^

Multiple drugs commonly used by the elderly could cause a decrease in LES pressure, leading to gastroesophageal reflux. The results of randomized controlled trials and systematic evaluations suggested that the use of non‐steroidal anti‐inflammatory drugs (NSAIDS) was associated with the onset of GERD.^35^, ^36^ In addition, nitrates, calcium antagonists, theophylline, tricyclic antidepressants, and benzodiazepines were all associated with the increased incidence of GERD.^37^

**Consensus opinion 2:** Esophageal motility disorders and reduced clearance ability in the elderly were important occurrence mechanisms of GERD in the elderly (recommendation grades: A+, 57%: A, 43%. Evidence level: Medium quality).

**Consensus opinion 3:** Esophageal hiatus hernia was an important influencing factor for GERD in the elderly (recommendation grades: A+, 48%; A, 48%; A−, 2%. Evidence level: High quality).

**Consensus opinion 4:** Chronic diseases were often comorbid in the elderly, which might be associated with the increased prevalence of GERD in the elderly (recommendation grades: A+, 28%; A, 51%; A−, 21%. Evidence level: Low quality).

**Consensus opinion 5:** Multiple drugs commonly used by the elderly could induce or worsen gastroesophageal reflux disease (recommendation grades: A+, 76%; A, 21%; A−, 2%. Evidence level: High quality).

## 4 DIAGNOSIS OF GERD IN THE ELDERLY

Heartburn and reflux are common and typical symptoms of GERD. Heartburn refers to a burning sensation in the posterior sternum, while reflux refers to the sensation of gastric contents flowing towards the pharynx and oral cavity; heartburn and reflux are the most common typical symptoms of GERD, but their incidence is lower in elderly patients with GERD than in the non‐elderly patients.^38^ A study with 264 elderly patients with GERD and 417 non‐elderly patients with GERD included showed that the incidences of heartburn and reflux in the elderly patients with GERD were as high as 52.3% and 42.4%, respectively, but both were significantly lower than those in the non‐elderly patients with GERD, and the incidences of heartburn and reflux in the non‐elderly patients with GERD were 62.1% and 68.6%, respectively.^33^

The severity of heartburn in elderly patients with GERD was not parallel to the degree of esophageal injury, the patients' symptoms were atypical or even asymptomatic, and they already had serious complications at the time of treatment.^39^ A study with 195 elderly patients with an average age of 74 years included showed that 50% of esophagitis patients had no symptoms of heartburn.^40^ Another large‐scale study with nearly 12,000 GERD patients included showed that the prevalence of severe erosive esophagitis gradually increased with aging, with 12% for the patients under 21 years old and 37% for the patients over 70 years old; in patients with severe esophagitis, the prevalence of severe heartburn symptoms gradually decreased with aging, with 82% for the patients under 21 years old and 34% for the patients over 70 years old.^41^ It can be seen that although the incidence of severe erosive esophagitis increases with age, the severity of heartburn is not parallel to it.

Some GERD patients do not have the typical symptoms of heartburn or reflux, but present atypical symptoms such as upper abdominal burning sensation, eructation, dysphagia, upper abdominal pain, or chest pain. A study with 264 elderly patients with GERD and 417 non‐elderly patients with GERD included showed that the incidences of upper abdominal burning sensation, eructation, dysphagia, upper abdominal pain, and chest pain in the elderly patients with GERD were 36.0%, 33.7%, 29.5%, 18.2%, and 17.8%, respectively; the incidences of upper abdominal burning sensation, dysphagia, upper abdominal pain, and chest pain in the non‐elderly patients with GERD were 24.5%, 13.4%, 12.5%, and 8.4%, respectively, all of which were significantly lower than those in the elderly patients with GERD.^33^ Another study that included 114 young patients with reflux esophagitis (<50 years old), 126 adult patients with reflux esophagitis (50–69 years old), 425 elderly patients with reflux esophagitis (70–84 years old), and 175 very elderly patients with reflux esophagitis (≥85 years old) showed that the incidence of dysphagia increased with age.^16^

**Consensus opinion 6:** Heartburn and reflux are still the typical and most common symptoms in elderly patients with GERD, but their incidences are lower than those in adult and young patients (recommendation grades: A+, 71%; A, 24%; A−, 5%. Evidence level: High quality).

**Consensus opinion 7:** The severity of heartburn in elderly patients with GERD cannot predict the degree of esophageal injury, and the severity of heartburn is not parallel to the severity of erosive esophagitis (recommendation grades: A+, 61%; A, 33%; A−, 6%. Evidence level: High quality).

**Consensus opinion 8:** Upper abdominal burning sensation, eructation, dysphagia, upper abdominal pain, chest pain, etc. are common atypical symptoms in elderly patients with GERD, and their incidences are higher than those in non‐elderly patients with GERD (recommendation grades: A+, 56%; A, 42%; A−, 2%. Evidence level: High quality).

Elderly patients with GERD may have chest pain; at the same time, the incidence of coronary artery disease in the elderly is significantly increased. Cardiogenic chest pain caused by coronary heart disease is also one of the common causes of chest pain in the elderly. To avoid the irreversible serious consequences that may be caused by delayed treatment of heart disease, for elderly patients with GERD with chest pain, it is necessary to first rule out cardiogenic chest pain. On the other hand, multiple studies have shown that gastroesophageal reflux is common in patients with coronary artery disease, with an incidence of 40% to 50%. Among them, chest pain in 40% to 70% of patients was directly related to gastroesophageal reflux.^42^, ^43^, ^44^, ^45^, ^46^ In addition, the most commonly used drugs for treating coronary artery disease, such as nitrates, calcium antagonists, and antiplatelet drugs, may induce or exacerbate GERD.^47^, ^48^ Given the above reasons, GERD should be suspected in the elderly patients with coronary artery disease patients and atypical or refractory angina^38^; previous studies have shown that proton pump inhibitors can effectively reduce chest pain and significantly improve quality of life in patients with coronary artery disease and GERD after acid suppressive therapy.^49^ In clinical practice, it is often difficult to distinguish between esophageal chest pain and cardiogenic chest pain in the elderly, and it requires experienced gastroenterologists and cardiologists to jointly evaluate the patient's condition and make accurate judgments.

The extraesophageal symptoms of GERD include sensation of foreign body in the throat, throat irritation, chronic cough, laryngopharyngeal reflux, hoarse voice, discomfort in the throat, reflux asthma, otitis media, and reflux sinusitis. The incidences of such symptoms in elderly patients with GERD are higher than those in non‐elderly patients.^38^, ^50^ A study of 222 elderly patients with GERD aged 65 and above showed that the incidences of chronic hoarseness, chronic cough, and wheezing were 26.9%, 23.4%, and 37.8%, respectively.^50^ Another study of 264 elderly patients with GERD and 417 non‐elderly patients with GERD showed that the incidence of extraesophageal symptoms in elderly patients with GERD was as high as 49.6%, higher than that in non‐elderly patients with GERD (38.1%).^33^ Idiopathic pulmonary fibrosis (IPF) is more common in people over 50 years old. Currently, literature reported that GERD is an independent risk factor for idiopathic pulmonary fibrosis, and GERD is highly prevalent in IPF.^51^, ^52^

**Consensus opinion 9:** If elderly patients with GERD have chest pain, first rule out cardiogenic chest pain, and pay attention to GERD in elderly patients with coronary artery disease (recommendation grades: A+, 71%; A, 29%. Evidence level: High quality).

**Consensus opinion 10:** The incidence of extraesophageal symptoms in elderly patients with GERD is high (recommendation grades: A+, 71%; A, 29%. Evidence level: High quality).

Considering that the incidence of digestive tract tumors increases with age and the GERD symptoms of the elderly are not consistent with the severity of the disease, and in combination with the fact that gastroscopy is widely carried out in China and the cost of examination is relatively low, it is recommended that gastroscopy should be the first choice for newly diagnosed elderly patients with gastroesophageal reflux, if there is no contraindication to endoscopic examination. A study in China showed that in 140 newly diagnosed patients over the age of 50 with typical reflux symptoms, the total detection rate of erosive esophagitis, Barrett's esophagus, peptic ulcer, and esophageal and gastric malignant tumors (one case of esophageal squamous cell carcinoma and one case of gastric adenocarcinoma) detected by gastroscopy was as high as 43.57%,^53^ proving the necessity of gastroscopy for such patients.

Gastroscopy is not only used for the differential diagnosis of digestive tract diseases in elderly patients with GERD, but also helps to accurately assess the severity of esophagitis in elderly patients with GERD and determine whether there are serious reflux‐related complications such as esophageal stenosis, Barrett's esophagus, and esophageal adenocarcinoma. Aging is an independent risk factor for severe erosive esophagitis in GERD patients.^54^, ^55^ Studies have shown that the incidences of erosive esophagitis and Barrett's esophagus were both significantly increased in GERD patients aged 60 and above.^56^ The incidences of reflux‐related esophageal ulcer, esophageal stenosis, and hemorrhage of digestive tract were also significantly increased in the elderly population.^16^, ^57^, ^58^ Therefore, gastroscopy is very important for assessing the severity of diseases in elderly patients with GERD.^59^

Esophageal dynamic reflux monitoring is the gold standard for GERD diagnosis, and commonly used methods include simple esophageal pH monitoring, multi‐channel intra‐luminal impedance combined with pH monitoring (MII‐pH), and wireless esophageal dynamic reflux monitoring. The sensitivity of simple esophageal pH monitoring for diagnosing GERD is 79% to 96%, and the specificity is 85% to 100%.^60^ MII‐pH monitoring can detect acid and non‐acid refluxes; distinguish liquid, gas, and mixed reflux; and provide information such as the reflux height, reflux speed, and reflux clearance time. It is currently a sensitive method for diagnosing GERD.^61^, ^62^, ^63^ Wireless esophageal dynamic reflux monitoring technology is used to monitor the pH value of the capsule fixed at the distal end of the esophagus through endoscopy. With the improvement of the process and the continuous reduction of the capsule volume, the monitoring time can reach 48 to 96 h, and the monitoring process is more in line with physiological conditions. At present, two types of catheterized esophageal dynamic reflux monitoring methods, namely, simple esophageal pH monitoring and MII‐pH monitoring, are still mainly used in China. Especially for patients only having atypical symptoms such as chest pain, respiratory system symptoms, or hoarse voice,^64^, ^65^, ^66^ and elderly patients with GERD who have no esophageal mucosal damage during gastroscopy and have poor treatment effects with acid suppressants, esophageal dynamic reflux monitoring can provide objective evidence of reflux, as the diagnostic basis for GERD.^66^, ^67^

**Consensus opinion 11:** For newly diagnosed elderly patients with symptoms of gastroesophageal reflux, it is recommended to first undergo gastroscopy (recommendation grades: A+, 60%; A, 33%; A−, 5%. Evidence level: High quality).

**Consensus opinion 12:** Dynamic esophageal reflux monitoring can serve as a detection method for objective evidence of reflux in elderly GERD patients with atypical symptoms, no esophageal mucosal damage, or poor response to treatment (recommendation grades: A+, 45%; A, 50%; A−, 5%. Evidence level: High quality).

## 5 TREATMENT OF GERD IN THE ELDERLY

Lifestyle intervention and dietary adjustment are the basic treatments for GERD. Multiple cohort surveys have shown that high fat foods were associated with an increase in GERD risk, while high fiber intake was associated with a decrease in GERD risk. The research results were not affected by age.^68^, ^69^, ^70^ Cohort studies and systematic reviews have also found that quitting smoking was beneficial for GERD treatment.^71^, ^72^ In clinical practice, it can be observed that abstinence from alcohol is helpful in avoiding reflux symptoms. Acid foods, high fat foods, and excessive or rapid consumption can all cause reflux.^73^ Gastroesophageal reflux symptoms in diabetes patients can be improved by controlling blood sugar.^74^ Proper use of positive pressure ventilation can help reduce nighttime reflux symptoms in patients with obstructive sleep apnea.

Multiple cohort surveys have shown that raising the head of the bed is effective in improving nighttime symptoms in GERD patients, and elderly people especially need to pay attention to fasting and drinking 2 to 3 h before going to bed at night. Due to the fact that napping is more common among elderly people and is more commonly associated with reflux compared to nighttime sleep, clear guidance on reflux prevention during naps should be given to elderly patients, with particular emphasis on the role of raising the head of the bed, such as using a wedge with a 20 degree angle. However, severe mucosal lesions often occur in elderly patients, and it is difficult to achieve mucosal healing solely by changing lifestyle.^75^

**Consensus opinion 13:** Lifestyle intervention and dietary adjustment are the basic treatments for GERD in the elderly (recommendation grades: A+, 40%; A, 58%; A−, 2%. Evidence level: Medium quality).

Acid suppressants are the preferred treatment for GERD. The ability of elderly people to secrete gastric acid does not decrease with age. The secretion of gastric acid in elderly people is similar to that in non‐elderly people. Even in elderly people over 85 years old, the gastric pH remains roughly normal at 24 h. Age alone does not affect the activity of hydrogen ions in the stomach.^76^ Proton pump inhibitors (PPIs) are still the main therapeutic drugs for GERD in the elderly, who are still highly sensitive to PPI treatment. Compared to typical symptoms of GERD such as heartburn and regurgitation, PPI is particularly effective for the more common extraesophageal symptoms of GERD in elderly patients.^77^ The efficacy of the new H^+^‐K^+^‐ATPase potassium competitive acid blocker (P‐CAB) is comparable to that of PPI. As a representative of the novel P‐CAB, vonoprazan competes with K^+^ to inhibit H^+^‐K^+^‐ATPase without the need for gastric acid induction activation, which is not affected by diet, and does not require taking before breakfast. It can be administered once a day, which may improve patient compliance. Therefore, P‐CAB is also recommended as the initial and maintenance treatment for GERD.^78^ In the entire age group, P‐CAB is not inferior to PPI in terms of mucosal healing rate and relief of regurgitation symptoms in esophagitis. P‐CAB may have a slight advantage in severe esophagitis.^79^ The Japan 2021 evidence‐based clinical practice guidelines for gastroesophageal reflux recommend P‐CAB (20 mg, once a day) for 4 weeks as the initial treatment for patients with severe esophagitis.^80^

The incidence rate of GERD in the elderly is high, and the acid suppression treatment that meets the needs of mucosal healing may be stronger. The recurrence rate of PPI in the elderly can be as high as 90%^81^ after withdrawal. A placebo‐controlled study showed that among patients aged 65 and above with erosive esophagitis, the mucosal healing rate in the Intention to Treat (ITT) analysis of PPI was 81%, while the mucosal healing rate in the Per Protocol (PP) analysis was 94%.^82^ Another prospective multicenter study in Japan showed a higher recurrence rate of erosive esophagitis in the elderly after discontinuing PPI.^83^ Maintenance treatment for the elderly can refer to the *Chinese expert consensus of gastroesophageal reflux disease in 2020*,^78^ and symptoms can be controlled through on‐demand treatment with PPI or P‐CAB. In particular, maintenance treatment for the elderly must consider its potential risks. PPI has a short half‐life, and GERD treatment in elderly patients does not require a reduction in PPI dosage. Pharmacokinetic studies have shown no difference in metabolism between middle‐aged and elderly individuals^84^; Patients with liver and kidney diseases do not need to adjust their dosage, as PPI is mainly metabolized by the liver. For severe liver damage, it is recommended to reduce the dosage.^85^ A survey based on 22,637 study subjects in Denmark showed that those who took PPIs, compared with those who did not take PPIs, were older (median age: 57 vs. 50 years old), had a higher obesity rate (16.7% vs. 13.1%), and had more concomitant diseases (35% vs. 15%).^86^, ^87^ In 2022, the National Medical Products Administration revised the PPI package inserts to include new adverse reactions or precautions such as diarrhea associated with *Clostridium difficile*, fracture, and hypomagnesaemia. The potential adverse reactions caused by long‐term use of PPIs in the elderly were mostly limited to retrospective and case–control studies, and although it was reported that they were associated with vitamin B12 deficiency,^88^ increased risk of *C. difficile* infection,^89^, ^90^ and poor absorption of minerals such as calcium and magnesium, there was a lack of evidence from high‐quality randomized controlled trials (RCTs).^91^ Patients with clear indications for PPI treatment should continue to receive treatment at the lowest effective dose. In a 24‐week double‐blind randomized phase 3 clinical study in Japan, the effectiveness and safety of vonoprazan were compared with those of lansoprazole in preventing peptic ulcers associated with low‐dose aspirin, 120 patients with dual antiplatelet therapy (low‐dose aspirin plus clopidogrel) combined with vonoprazan or lansoprazole were enrolled, and no cardiovascular events occurred during the observation period.^92^

**Consensus opinion 14:** PPIs and P‐CABs were the preferred treatment drugs for GERD in the elderly (recommendation grades: A+, 52%: A, 43%; A−, 5%. Evidence level: High quality).

**Consensus opinion 15:** GERD in the elderly was chronic recurrent and often required maintenance treatment (recommendation grades: A+, 22%; A, 68%; A−, 10%. Evidence level: Medium quality).

**Consensus opinion 16:** Attention needed to be paid to the potential risks and drug interactions of long‐term acid suppressive therapy in the elderly (recommendation grades: A+, 24%; A, 57%; A−, 19%. Evidence level: Low quality).

Regarding to prokinetic drugs, it was believed in US guidelines that their evidence for GERD treatment was limited, and it was also not recommended in the guidelines and expert consensus from China, Japan, and South Korea that prokinetic drugs should be used alone for GERD patients, but it was believed that prokinetic drugs used in combination with PPIs could help improve symptoms in some GERD patients. A meta‐analysis of 14 RCTs with 1437 GERD patients included in China showed that compared with PPI monotherapy, the combination of prokinetic drugs with PPIs could significantly improve symptoms.^93^ A system evaluation and meta‐analysis of 16 RCTs with 1446 GERD patients included in South Korea showed that compared with PPI monotherapy, the combination of prokinetic drugs with PPIs could significantly improve the overall symptoms of refractory GERD and non‐refractory GERD.^94^ A RCT with 70 patients with refractory GERD with a median age of >60 years included in Japan showed that compared with PPI monotherapy, the combination of prokinetic drugs with PPIs could significantly improve the symptoms of refractory non‐erosive reflux disease (NERD) patients.^95^ Commonly used prokinetic drugs include mosapride, cinitapride, itopride, etc. In the application of prokinetic drugs in the elderly, special attention should be paid to their adverse reactions (such as extrapyramidal reactions and prolonged Q‐T interval on electrocardiogram) and drug interactions.^96^ Itopride was metabolized by flavin‐containing monooxygenase instead of CYP450 enzyme, with minimal drug interactions and no affinity with 5‐hydroxytryptamine 4 receptors, making it less prone to cardiovascular adverse events caused by prolonged Q‐T interval.^97^, ^98^

**Consensus opinion 17:** The combination of prokinetic drugs with acid suppressants could help improve GERD symptoms (recommendation grades: A+, 54%; A, 34%; A−, 12%. Evidence level: Medium quality).

Fundoplication has been proven to effectively improve LES pressure, reduce esophageal acid exposure time, and improve the quality of life score of GERD patients, with high safety.^99^, ^100^ It is currently recommended as an anti‐reflux surgery method in multiple GERD‐related guidelines at home and abroad. Laparoscopic fundoplication (LF) is superior to open fundoplication and has become the current standard anti‐reflux surgery.^78^ For GERD patients with clear and objective evidence of reflux, especially those with severe reflux esophagitis (Los Angeles grade C or D), large hiatus hernia, reluctance to long‐term use of PPI treatment, and/or refractory reflux, experienced surgeons may be chosen to perform LF. There were currently no high‐quality data to confirm the effectiveness of surgical treatment for extraesophageal symptoms of GERD, so doctors should be particularly cautious when recommending such treatment to patients with laryngopharyngeal reflux (LPR) and other extraesophageal reflux symptoms.^72^

Multiple studies have confirmed the effectiveness and safety of LF in elderly patients with GERD. Fei et al.^101^ consecutively included 620 patients undergoing LF in a prospective non‐randomized cohort study published in 2013, which showed that 90% of the patients in the elderly group (≥65 years old) had significantly improved postoperative reflux symptoms while 90% in the young group (*p* > 0.05), the incidence of postoperative dysphagia was 3% while 3% in the young group (*p* > 0.05), and the incidences of other symptoms such as abdominal distension, early satiety, and chest pain were also not different from those in the young group.^101^ A retrospective study published by Tedesco et al.^102^ in 2006 also showed that there were no statistically significant differences in LF duration, intraoperative and postoperative complications, and hospital stay between the group with patients ≥65 years old and the group with patients <65 years old, and 90% of the patients had improvement in heartburn symptoms and did not require long‐term dependence on PPIs in the both groups. However, it should be noted that the above studies should include elderly patients with GERD who have been screened by surgeons and are believed to be able to tolerate surgery. In summary, for elderly patients with GERD, anti‐reflux surgery should be considered, based on a thorough assessment of their overall health status, expected lifespan and anesthesia risks, combined with their own wishes and on the premise of meeting the indications.

Before choosing the surgery, it is also necessary to consider the long‐term effectiveness of LF. A recent population‐based retrospective cohort study from Sweden included 2655 patients who underwent LF from 2005 to 2014, with a median follow‐up time of 5.6 years, among whom 470 (17.7%) experienced reflux recurrence, of whom 393 (83.6%) required long‐term anti‐reflux drug therapy, and 77 (16.4%) underwent secondary anti‐reflux surgery; the results showed that the risk factors for GERD symptom recurrence after LF were the age ≥61 years (HR = 1.41, 95% CI: 1.10–1.81), females (HR = 1.57, 95% CI: 1.29–1.90), and comorbidities (Charlson comorbidity index ≥1) (HR = 1.36, 95% CI: 1.13–1.65).^103^ Therefore, for elderly patients with GERD, if they are ready to receive LF treatment, it is also necessary to consider that there may be a higher possibility of symptom recurrence after the surgery than that in young people.

In the past 20 years, multiple new technologies for endoscopic treatment of GERD have emerged. Currently, the most widely used ones are endoscopic radiofrequency therapy and transoral incisionless fundoplication (TIF), and peroral endoscopic cardial constriction (PECC) and endoscopic anti‐reflux mucosectomy (ARMS) are still under exploration. Endoscopic treatment enhances the anti‐reflux function of the gastroesophageal junction through endoscopic treatment, which, compared with surgical treatment, may have advantages in the treatment of GERD in the elderly due to its more minimally invasive nature. Endoscopic treatment is suitable for mild gastroesophageal reflux disease patients with clear evidence of reflux or effective PPIs. It is not suitable for patients with hiatus hernia above 2 cm, grades C and D esophagitis, esophageal stenosis, or long‐segment Barrett's esophagus. Before endoscopic treatment, esophageal high‐resolution manometry should be performed to exclude achalasia and other esophageal motility disorders.^72^, ^78^ A meta‐analysis (2469 patients in total) which include 4 randomized controlled studies, 23 cohort studies, and 1 registry study, showed that radiofrequency (RF) therapy significantly reduced acid exposure time, improved heartburn symptoms and quality of life in GERD patients, and reduced the use of PPIs.^104^ At present, RF therapy has been proven to have good safety, with a complication rate of less than 1%, and is mainly characterized by mild mucosal erosions and lacerations.^104^ TIF has not been widely carried out in China and only partial clinical trials have been conducted. A systematic review and meta‐analysis of the use of TIF in the treatment of refractory gastroesophageal reflux in 2018 showed that TIF significantly improved the health‐related quality of life and DeMeester score related to gastroesophageal reflux, enabling 89% of patients to discontinue PPIs.^105^ In recent years, another meta‐analysis has shown that TIF cannot reduce acid exposure and PPI dosage during long‐term follow‐up.^106^ The incidence of TIF serious complications ranges from 2.4% to 7.18%, commonly including subcutaneous emphysema, perforation, and hemorrhage.^105^, ^106^

A systematic review and network meta‐analysis published in 2021 included 516 patients from 10 studies and compared the efficacy of endoscopic anti‐reflux therapy and PPIs on GERD. Some studies included some elderly cases, and the results showed that in the short term (6 months), TIF and RF therapy had no difference in improving heartburn symptoms and health‐related quality of life (HRQL) scores in patients with GERD, both of which were superior to PPIs. For increasing LES pressure, TIF was superior to RF and PPIs, but for reducing acid exposure time (AET), TIF was inferior to PPIs.^107^ However, the conclusions drawn need further validation given the small number of included study cases and poor homogeneity.

The above endoscopic anti‐reflux therapy currently lacks large‐scale research data in the elderly population. It is recommended to carefully evaluate the indications based on a comprehensive evaluation of the systemic disease situation, life expectancy, and tolerance to endoscopy and anesthesia in elderly patients, combined with the results of high‐resolution esophageal manometry and pH impedance monitoring, endoscopic GERD complications, and past drug treatment effects.

**Consensus opinion 18:** Laparoscopic fundoplication for GERD is safe and effective and can be used in elderly patients with GERD. However, it is necessary to strictly collect indications, carefully weigh risks and benefits of surgical treatment, and consider long‐term efficacy (recommendation grades: A+, 33%: A, 26%; A, 55%. Evidence level: High).

**Consensus opinion 19:** Endoscopic anti‐reflux therapy can be used in elderly patients with mild GERD with clear evidence of reflux and effective PPIs after comprehensive evaluation and careful screening (recommendation grades: A+, 26%: A, 49%; A−, 23%. Evidence level: Low quality).

## 6 DIAGNOSIS AND TREATMENT OF EXTRAESOPHAGEAL SYMPTOMS IN ELDERLY PATIENTS WITH GERD

GERD's extraesophageal symptoms include asthma, chronic cough, chronic pharyngitis, etc. GERD is common in asthma patients, with 34% to 89% of patients having GERD. GERD is considered to be a potential trigger for asthma.^108^, ^109^, ^110^, ^111^ Pharyngeal reflux refers to the reflux of gastric contents into the pharynx, leading to symptoms related to the pharynx. A considerable portion of symptoms such as chronic pharyngitis, chronic cough, hoarseness, dry cough, clear throat, globus hystericus, and mild dysphagia can be caused by pharyngeal reflux.^112^, ^113^, ^114^, ^115^ However, GERD‐related throat symptoms are not specific, and non‐reflux factors need to be excluded before diagnosis of reflux‐related diseases. The corresponding specialist can evaluate whether there are other diseases such as throat or lung diseases.^116^ The mechanisms by which GERD causes extraesophageal symptoms include direct stimulation of the laryngeal and pharyngeal tissues by reflux gastric contents,^117^, ^118^ vagal reflex induced, airway hyperresponsiveness induced by reflux,^119^, ^120^, ^121^, ^122^ and microaspiration of gastric contents into the upper airway.^123^

Multiple clinical trials have shown that PPI treatment can improve asthma and throat reflux symptoms in patients with typical reflux symptoms.^124^, ^125^ However, for patients without typical reflux symptoms, PPI treatment did not improve asthma outcomes and throat reflux symptoms.^126^, ^127^ The efficacy of acid suppressive therapy on GERD‐related extraesophageal symptoms is still controversial.^128^, ^129^

In the elderly population, there is currently a lack of large‐scale clinical studies on the treatment of elderly GERD patients with extraesophageal symptoms. The results of small‐scale studies have shown that PPIs are effective in the treatment of elderly patients with laryngopharyngeal reflux symptoms. A prospective multicenter study included 264 patients with laryngopharyngeal reflux. The results showed that different age groups showed significant improvement in both reflux symptoms and laryngopharyngeal reflux symptoms after treatment with lansoprazole, and there was no significant difference in the degree of improvement between elderly and young people.^130^ A clinical study in China included 66 GERD patients with extraesophageal symptoms (including cough, pharyngitis, and asthma) who were treated with esomeprazole for 8 weeks. The study results showed that both elderly and young patients had significant improvement in acid exposure and symptoms by gastroscopy after treatment, and there was no statistically significant difference between the two groups, indicating that PPI has a better therapeutic effect on elderly patients with reflux esophagitis and extraesophageal symptoms, and there was no significant difference compared with young patients.^131^ Therefore, for elderly patients with typical reflux symptoms such as asthma, chronic cough, and chronic pharyngitis, PPI can be considered for diagnostic treatment.

**Consensus opinion 20:** GERD is a possible cause of asthma, chronic cough, and chronic pharyngitis (recommendation grades: A+, 50%; A, 45%; A−, 2%. Evidence level: Medium quality).

**Consensus opinion 21:** For elderly patients with asthma, chronic cough, and chronic pharyngitis who are suspected of gastroesophageal reflux, PPIs can be considered for diagnostic treatment (recommendation grades: A+, 52%; A, 39%; A−, 9%. Evidence level: Low quality).

## 7 DIAGNOSIS AND TREATMENT OF REFRACTORY GERD IN THE ELDERLY

The definition of refractory GERD is still controversial. According to the Chinese Expert Consensus on GERD, refractory GERD is defined as those who have no significant improvement in heartburn or reflux symptoms after 8 weeks of double dose PPI treatment.^78^ At present, there is no study on the statistics of the incidence of refractory GERD in the elderly population. There are many factors that can cause refractory GERD, including failure to strictly follow the medication timing of PPI and poor compliance to PPI treatment,^132^, ^133^, ^134^ residual acid reflux^135^ after receiving PPI treatment, weak or non‐acidic reflux, hypersensitive reflux,^136^ and functional heartburn,^137^ all of which can cause refractory GERD.

For elderly patients with poor conventional treatment results, attention should be paid to visceral hypersensitivity and psychological factors. Some domestic researchers believe that psychological factors in the elderly are important influencing factors for refractory GERD.^138^ The incidence of hypersensitive esophagus, functional heartburn, and non‐cardiogenic chest pain in the elderly is not low.^139^ The hypersensitive esophagus is associated with mental states (including anxiety and depression) and sleep disorders, which can alter esophageal sensitivity. Antidepressants may be beneficial for symptoms related to esophageal visceral hypersensitivity in GERD patients. Studies have reported that esophageal pain thresholds increased by 7% to 37% after antidepressant therapy. Antidepressant therapy reduced functional chest pain over a range from 18% to 67% and reduced heartburn over a range of 23% to 61%.^140^

It is recommended to improve upper gastrointestinal endoscopy, esophageal impedance pH monitoring, and esophageal manometry for patients with refractory GERD, and analyze the reasons for the poor treatment effect of PPIs. Upper gastrointestinal endoscopy can help exclude other esophageal and gastric diseases, such as gastrointestinal tumors and achalasia, and esophageal biopsy should be performed to exclude eosinophilic esophagitis and other esophagitis. In addition to detecting acidic reflux, esophageal impedance pH monitoring can also detect non‐acidic reflux,^141^, ^142^ and can monitor all reflux events including acid, weak acid, non‐acid, and gas reflux, helping to distinguish between hypersensitive esophagus and functional heartburn.^143^, ^144^ For patients with dysphagia and reflux, as well as before invasive anti‐reflux therapy, it is recommended to undergo esophageal manometry to rule out esophageal motility disorders.^145^, ^146^ For patients who are not qualified to undergo esophageal impedance pH monitoring, the type of PPI can be empirically changed or P‐CAB can be used.^147^

For patients with persistent acidic reflux detected by esophageal impedance pH monitoring, a bedtime dose of an H_2_‐receptor antagonists is recommended.^148^ For patients with esophageal impedance pH monitoring showing non‐acidic reflux and related symptoms, it is recommended to try using baclofen. For refractory GERD patients with normal esophageal impedance pH monitoring results (esophageal hypersensitivity or functional heartburn), it is recommended to try using pain regulators such as tricyclic antidepressants, serotonin norepinephrine reuptake inhibitors, selective serotonin reuptake inhibitors, or trazodone.^140^ For patients who continue to experience GERD symptoms and have delayed gastric emptying after PPI treatment, it is recommended to use prokinetic drugs for treatment. There is currently a lack of high‐quality clinical studies on drug treatment for elderly patients with refractory GERD.

After comprehensive and meticulous examination to exclude other causes, if there is indeed evidence of symptomatic reflux for refractory GERD that has failed drug treatment, anti‐reflux surgery is feasible.^78^ Anti‐reflux surgery includes therapeutic endoscopy (RF therapy and peroral incisionless fundoplication) and surgical surgery (laparoscopic fundoplication). A randomized controlled study screened 366 patients with refractory heartburn and ultimately included 78 patients with PPI‐refractory heartburn. The results showed that the incidence of treatment success with laparoscopic fundoplication, active medical treatment (PPI plus baclofen, combined with desipramine according to symptoms), and control medical treatment (PPI plus placebo) were 67%, 28%, and 12%, respectively.^149^ There is currently a lack of research data on anti‐reflux surgery in elderly patients with refractory GERD.

**Consensus opinion 22:** There are many factors that cause refractory GERD, and poor compliance with PPI treatment is an important reason for refractory GERD. For elderly patients with poor conventional treatment results, attention should be paid to visceral hypersensitivity and psychological factors (recommendation grades: A+, 26%; A, 60%; A−, 14%. Evidence level: Low quality).

**Consensus opinion 23:** It is recommended to improve upper gastrointestinal endoscopy, esophageal impedance pH monitoring, and esophageal manometry for patients with refractory GERD; if there is indeed evidence of symptoms‐related reflux in refractory GERD patients who have failed drug treatment, anti‐reflux endoscopy and surgical therapy are feasible (recommendation grades: A+, 19%; A, 53%; A−, 21%. Evidence level: Medium quality).

## 8 MANAGEMENT OF GERD COMPLICATIONS IN THE ELDERLY

Barrett's esophagus is defined as a condition in which the junction line between esophageal squamous epithelium and columnar epithelium moves upward relative to the gastroesophageal junction under endoscopy, and it is histologically confirmed that normal stratified squamous epithelium is replaced by metaplastic columnar epithelium. Barrett's esophagus includes three histological types: gastric fundus gland mucosal metaplasia, cardiac gland mucosal metaplasia, and intestinal mucosal epithelial metaplasia. Barrett's esophagus with intestinal mucosal epithelial metaplasia has a higher risk of developing esophageal adenocarcinoma.^78^ The incidence of GERD complications gradually increases with age. A study has confirmed that the incidence of erosive esophagitis and Barrett's esophagus is significantly higher in patients over 60 years (81%) compared to young people (47%), and the incidence of Barrett's esophagus is significantly higher in elderly people (25%) compared to young people (15%).^56^ The diagnosis of Barrett's esophagus relies on endoscopy and pathological examination, which can determine the presence of Barrett's esophagus, clarify its histological type, and determine whether it is accompanied by dysplasia, which helps to develop follow‐up and treatment strategies. The annual risk of developing esophageal adenocarcinoma in patients with nondysplastic Barrett's esophagus is 0.2% to 0.5%, whereas in patients with low‐grade dysplasia (LGD) it increases to 0.7% to 1% and high‐grade dysplasia (HGD) increases to 7% to 8%.^150^

Endoscopic and tissue biopsy pathological examination monitoring of Barrett's esophagus is currently the only relatively sufficient follow‐up method.^116^ For Barrett's esophagus without dysplasia, the recommended follow‐up interval in the United States, UK, and Asia‐Pacific consensuses is 3 to 5 years. The current consensuses have not yet established different follow‐up intervals based on age, but it is important to note that in endoscopic screening and follow‐up for the elderly, the most important point is to measure life expectancy and the benefits and risks of invasive endoscopic examination and treatment. In a study, out of 4252 male veterans aged ≥65 diagnosed with Barrett's esophagus, 32% had limited life expectancy at the time of diagnosis, and 26% died within 4 years after diagnosis,^151^ indicating the importance of evaluating life expectancy when developing follow‐up and treatment strategies in elderly patients.

Patients with Barrett's esophagus with low‐grade dysplasia should be closely followed up or undergo endoscopic resection or ablation treatment; for patients with Barrett's esophagus with high‐grade dysplasia, endoscopic resection may be considered, but a comprehensive evaluation of the depth of lesion infiltration and the risk of lymph node metastasis is required. Surgery may be considered for those who do not meet the indications for endoscopic treatment.^116^ When formulating endoscopic treatment strategies for elderly patients, it is necessary to measure the risks and benefits, taking into account factors such as patient complications, prognosis, treatment willingness, and compliance. Additionally, due to more common concomitant medications among elderly patients, it is necessary to consider the risks caused by the withdrawal of anticoagulant and anti‐aggregation drugs before surgery, so as to develop comprehensive treatment strategies.

**Consensus opinion 24:** Barrett's esophagus is an important complication of GERD. The incidence of Barrett's esophagus is higher in the elderly, and the diagnosis requires endoscopic and pathological examination (recommendation grades: A+, 55%: A, 35%; A−, 10%. Evidence level: High quality).

**Consensus opinion 25:** Patients with Barrett's esophagus with dysplasia and esophageal adenocarcinoma should undergo follow‐up, endoscopic, or surgical treatment, but follow‐up and treatment strategies should be developed based on a comprehensive evaluation of the expected lifespan, complications, and other conditions of the elderly (recommendation grades: A+, 40%: A, 50%: A−, 10%. Evidence level: High quality).

Prolonged and uncured severe reflux esophagitis may lead to the occurrence of esophageal stenosis, which is a complication of severe reflux esophagitis. Esophageal stenosis has a higher incidence in the elderly, which may be related to the atypical symptoms in the elderly, leading to the persistence of long‐term asymptomatic reflux. Esophageal stenosis may be asymptomatic or manifest as difficulty in swallowing. Esophageal stenosis can be diagnosed through upper gastrointestinal barium radiography, lipiodol angiography, or endoscopy. Esophageal stenosis is most common in the lower segment of the esophagus, usually adjacent to the dentate line and adjacent to the area of erosive esophagitis. If stenosis occurs in an atypical location or has atypical features, biopsy is required to exclude the possibility of malignancy. The main treatment method for esophageal stenosis is balloon dilation or bougienage, but there is a certain relapse rate after surgery. Studies have shown that long‐term acid suppressive therapy is needed after surgery to reduce the relapse rate.^152^, ^153^ There is currently a lack of high‐quality clinical studies on esophageal stenosis in the elderly.

**Consensus opinion 26:** Esophageal stenosis is a complication of severe reflux esophagitis, with a higher incidence in the elderly. Patients with esophageal stenosis still need to maintain acid suppressive therapy after dilation treatment (recommendation grades: A+, 49%: A, 42%: A−, 7%. Evidence level: Medium quality).

## AUTHOR CONTRIBUTIONS

Initiate and organization of this consensus: Xu Le, Zheng Songbai. Writing the initial draft: Liu Fangxu, Zhang Pan, Chen Dan, Wu Xi, Xu Xue, Li Wenbin, Shi Jihua, Luo Qingfeng. Preparation and presentation of the published work: Liu Fangxu. Translator: Li Wenbin. Critical review and revision: the Expert Group of the Chinese Expert Consensus on diagnosis and management of gastroesophageal reflux disease in the elderly.

## FUNDING INFORMATION

This study was supported by National High Level Hospital Clinical Research Funding (BJ‐2022‐088).

## CONFLICT OF INTEREST STATEMENT

Zheng Songbai is the Editorial Board member of the journal and was excluded from the peer‐review process and all editorial decisions related to the publication of this article. Other authors have nothing to disclose.

## ETHICS STATEMENT

Not applicable.

## APPENDIX 1 EXPERT GROUP MEMBERS (LIST IN ALPHABETIC ORDER BY THE LAST NAME IN CHINESE PINYIN)

### 1.1

Chen Minhu (Department of Gastroenterology, The First Affiliated Hospital of Sun Yat‐sen University); Chen Xinyu (Department of Gastroenterology, Zhejiang Hospital); Ding Shigang (Department of Gastroenterology, Peking University Third Hospital); Ding Xiping (Department of Geriatrics, Anhui Provincial Hospital); Hou Xiaohua (Department of Gastroenterology, Wuhan Union Hospital); Huang Xiaoli (Department of Geriatrics, West China Hospital, Sichuan University); Ji Rui (Department of Gastroenterology, The First Hospital of Lanzhou University); Jiang Hua (Department of Geriatrics, East Hospital Affiliated to Tongji University); Li Jingnan (Department of Gastroenterology, Peking Union Medical College Hospital); Li Peng (Department of Gastroenterology, Beijing Friendship Hospital); Liu Ruixue (Department of Gastroenterology, The People's Hospital of Liaoning Province); Liu Shixiong (Department of Geriatrics, The First Hospital of Lanzhou University); Liu Zhijun (Pharmacy Department, Beijing Anzhen Hospital); Long Limin (Department of Geriatrics, The Second Xiangya Hospital of Central South University); Luo Qingfeng (Department of Gastroenterology, Beijing Hospital, National Center of Gerontology); Miao Lin (Department of Gastroenterology, The Second Affiliated Hospital of Nanjing Medical University); Ruan Jigang (Department of Gastroenterology, General Hospital of Ningxia Medical University); Shao Yun (Department of Elderly Gastroenterology, Jiangsu Province Hospital); Shi Liping (Department of Elderly Gastroenterology, Shaanxi Provincial People's Hospital); Sun Xiaohong (Department of Gastroenterology, Peking Union Medical College Hospital); Tang Chengwei (Department of Gastroenterology, West China Hospital, Sichuan University); Wan Jun (Department of Gastroenterology, Second Medical Center, Chinese PLA General Hospital); Wang Fengyun (Department of Gastroenterology, Xiyuan Hospital, China Academy of Chinese Medical Sciences); Wang Gangshi (Department of Gastroenterology, Second Medical Center, Chinese PLA General Hospital); Wang Jingtong (Department of Gastroenterology, Peking University People's Hospital); Wang Ruiling (Department of Gastroenterology, PLA Rocket Force Characteristic Medical Center); Wen Shujun (Department of Gastroenterology, The Second Hospital of Tianjin Medical University); Wu Jimin (Center for Gastroesophageal Reflux Disease, PLA Rocket Force General Hospital); Wu Jing (Department of Gastroenterology, Beijing Friendship Hospital); Xiao Yinglian (Department of Gastroenterology, The First Affiliated Hospital of Sun Yat‐sen University); Xu Le (Department of Gastroenterology, Beijing Hospital, National Center of Gerontology); Xu Lishu (Department of Gastroenterology, Guangdong Provincial People's Hospital); Xu Shiping (Department of Gastroenterology, Chinese PLA General Hospital); Yao Jianfeng (Department of Gastroenterology, Huadong Hospital); Yao Ping (Department of Gastroenterology, The First Affiliated Hospital of Xinjiang Medical University); Yin Tiejun (Department of Geriatrics, Wuhan Tongji Hospital); Yu Pulin (Beijing Hospital, National Center of Gerontology); Zhang Hangxiang (Department of Geriatrics, Xijing Hospital of Air Force Military Medical University); Zhang Weisan (Department of Geriatrics, Tianjin Medical University General Hospital); Zhang Yijun (Department of Gastroenterology, General Hospital of Southern Theatre Command of PLA); Zhao Li (Department of Gastroenterology, Beijing Hospital, National Center of Gerontology); Zheng Songbai (Department of Gastroenterology, Huadong Hospital Affiliated to Fudan University); Zhong Bihui (Department of Gastroenterology, The First Affiliated Hospital of Sun Yat‐sen University); Zhou Bingxi (Department of Gastroenterology, Henan Provincial People's Hospital); Zhou Liya (Department of Gastroenterology, Peking University Third Hospital); Zou Duowu (Department of Gastroenterology, Ruijin Hospital).
